# Robotic dual-docking surgery for para-aortic lymphadenectomy in endometrial cancer: a prospective feasibility study

**DOI:** 10.1007/s10147-024-02635-8

**Published:** 2024-12-21

**Authors:** Shintaro Yanazume, Hiroaki Kobayashi, Takashi Ushiwaka, Shinichi Togami, Masaki Kamio

**Affiliations:** Department of Obstetrics & Gynecology, Faculty of Medicine, Kagoshima University, 8-35-1 Sakuragaoka, Kagoshima, 890-8520 Japan

**Keywords:** Endometrial cancer, Robotic surgery, Dual-docking, Para-aortic lymphadenectomy

## Abstract

**Background:**

The standard for robotic para-aortic lymphadenectomy has not been fully established. Para-aortic lymphadenectomy performed by sharing the same ports with pelvic procedures, a procedure known as dual-docking surgery, can be performed using the latest robotic system. We prospectively examined the ability of standardized dual-docking robotic surgery in endometrial cancer patients.

**Methods:**

This study prospectively verified the feasibility and safety of dual-docking robotic surgeries performed between March 2017 and December 2021. The laterally placed ports were aligned with the umbilicus. Primary outcome was the surgical completion rate; secondary outcomes were blood loss, operative time, unexpected port placement, conversion, complications, length of hospital stay, and survival.

**Results:**

Most patients (14/15, 93%) underwent surgery using our methods without additional port placements, and one patient was converted to laparotomy. Median blood loss was 162 mL (range: 20–685 mL). Median operative time was 183 and 206 min in the upper and lower abdomen. Median number of resected para-aortic lymph nodes was 19 (range: 6–29), and pelvic lymph nodes was 28 (range: 15–42). Although there was no difficulty in moving the forceps intraoperatively, major complications including vessel injury, and pelvic abscesses were observed. The lateral ports could be placed 6–10 cm apart in patients with any range of body type.

**Conclusion:**

Dual-docking surgery for endometrial cancer has the potential to be a standard procedure for robotic endometrial cancer surgery, although a greater number of cases are needed to acquire proficiency.

**Supplementary Information:**

The online version contains supplementary material available at 10.1007/s10147-024-02635-8.

## Introduction

Endometrial cancer is the most common gynecological malignancy in Japan and other countries. The number of patients was 11 085 in 2016, which was an approximately threefold increase since 2003 [1]. Simple abdominal hysterectomy, bilateral salpingo-oophorectomy, and lymph node assessment are standard surgeries for uterine-confined endometrial carcinoma [2, 3]. Although the therapeutic strategy for lymphadenectomy in low-risk endometrial cancer has produced no survival benefits, systemic para-aortic lymphadenectomy has provided such benefits in patients with intermediate-to high-risk endometrial cancer (hazard ratio 0.48, 95% CI 0.29–0.83; *p* = 0.0049) compared to pelvic lymphadenectomy alone in a SEPAL study [4].

Studies regarding the safety and prognosis of robotic minimally invasive surgery for endometrial cancer have been reported since 2008 [5–7]. A review comparing robotic surgery with laparoscopy or open laparotomy in endometrial cancer surgery identifies the positive outcomes in terms of safety and prognosis [8]. Robotic surgery has a shorter hospital stay, less estimated blood loss, and lower complication rates than other surgeries [6, 8–11]. Furthermore, minimally invasive techniques are widely used for uterine-confined endometrial carcinoma [1, 3].

In high-risk endometrial cancer, a wide range of surgeries may be needed, addressing areas from the upper abdomen (including the para-aortic lymph nodes and appendix when the high-risk histologic type) to the lower abdomen. Robotic para-aortic lymphadenectomy is performed by sharing the ports for pelvic procedures; this is called dual-docking surgery. Surgery using the same ports for both areas is reasonable practice, and newer robotic improvements have the potential to completely achieve dual-docking surgery for endometrial cancer treatment. Generally, additional port placement on the upper pubis is required when implementing dual-docking surgery in the Da Vinci S or Si systems (Intuitive Surgical Inc., Sunnyvale, CA, USA), but not around the umbilicus [12–15]. It is troublesome to create ports on the upper abdominal wall for pelvic cavity surgery after closing the lower abdominal wall wound, as it may increase subcutaneous emphysema. Trocar placement is the most important factor for the success of dual-docking surgery, but the ports are not always placed suitably for various types of habitus. When performing robotic surgery on both the lower and upper abdomen, the point of aorta bifurcation is the suitable position for endoscope entry [16]. Although the umbilicus is most frequently used for introducing an endoscope port because of its location on the aortic bifurcation, the location of the umbilicus changes greatly depending on the habitus and posture, especially in obese women [17, 18]. Limited previous reports describe the successful application of ports at the level of the superior iliac spine for dual-docking endometrial cancer surgery using the Da Vinci Si system [16]. In the latter report, the rotation of the robotic column and movement of the patient’s bed were required to change the surgical range from the upper abdomen to the lower abdomen, which is called double-side docking. In contrast, our dual-docking allows for simple rotation of the boom without moving the patient cart along with direct docking of the ports.

There has been little detailed information regarding dual-docking surgery with ports set at the standard level of the umbilicus using the Da Vinci Xi system. The present study prospectively examined the ability of standardized dual-docking robotic surgery in endometrial cancer patients in the Japanese population.

## Patients and methods

### Study design

This prospective, single-center study was designed to verify the feasibility and safety of dual-docking surgery performed using the Da Vinci Xi surgical system at Kagoshima University Hospital for patients with endometrial cancer. The patients were recruited between March 2017 and December 2021. The standardization of dual-docking surgery with laterally aligned ports placed at the level of the umbilicus for both upper and lower abdominal surgery was investigated. The primary outcome was the completion rate of upper or lower abdominal surgery after dual-docking surgery. The secondary outcomes were blood loss, operative time, unexpected port placement, conversion to laparotomy, intraoperative complications, length of hospital stay, and survival.

The inclusion criteria were as follows: (1) poorly differentiated endometrioid carcinoma or a high-risk histologic subtype; serous carcinoma, clear cell carcinoma, or carcinosarcoma with suspected International Federation of Gynecology and Obstetrics (FIGO, 2008) stage 1A endometrial cancer; (2) preoperative FIGO stage IB or stage II endometrial cancer; and (3) written informed consent from patients aged ≥ 20 years.

Exclusion criteria included: (1) simultaneous or metachronous double cancers; (2) pregnancy, postpartum within 28 days, and breastfeeding; (3) complicated with a serious disease; (4) mental disorder; (5) glaucoma; and (6) untreated cerebral aneurysm.

Operative time was defined as the time between the start of the skin incision and the closure of the incision following the removal of all ports. Rotation time was defined as the time between finishing the upper abdominal console and starting the lower abdominal console. Surgical complications were assessed using the Clavien–Dindo Classification v.2.0. All dual-docking surgeries were performed by a single gynecologic oncologist with a Class A Robo Doc certificate from the Japan Robotic Surgery Society.

This trial was conducted in accordance with the Declaration of Helsinki, and the protocol was approved by the institutional review board (170,152, 180,230) before patient enrollment. Despite there being a conflict of interest between the researcher and Intuitional Surgical Inc., this research was planned independently by the researcher, and the company was not involved in the planning, implementation, analysis, and reporting of this research; therefore, it did not affect the interpretation of results.

### Surgical technique details

Preoperative evaluations were the same as those of open laparotomy, which included endometrial curettage, endocervical curettage, computed tomography of the thorax to the lower abdomen, and pelvic magnetic resonance imaging. Intraocular pressure measurements were performed before surgery.

Standard robotic surgical techniques included peritoneal cytology, hysterectomy, bilateral salpingo-oophorectomy, para-aortic lymphadenectomy, pelvic lymphadenectomy, and omentectomy/appendectomy (if indicated), similar to open laparotomy. Para-aortic lymphadenectomy was defined as a resection up to the level of the left renal vein. If the surgery was difficult to complete due to bleeding or complications, the procedure was promptly converted to open laparotomy. If safety could not be maintained with our methods, ports were constructed in the lower and upper abdomen as appropriate. Postoperative chemotherapy was administered to patients with intermediate-to-high-risk endometrial cancer according to the same postoperative pathological diagnosis as laparotomy according to the Japan Society of Gynecologic Oncology Guidelines [2].

### Procedures for dual-docking surgery

**Fig. 1:**
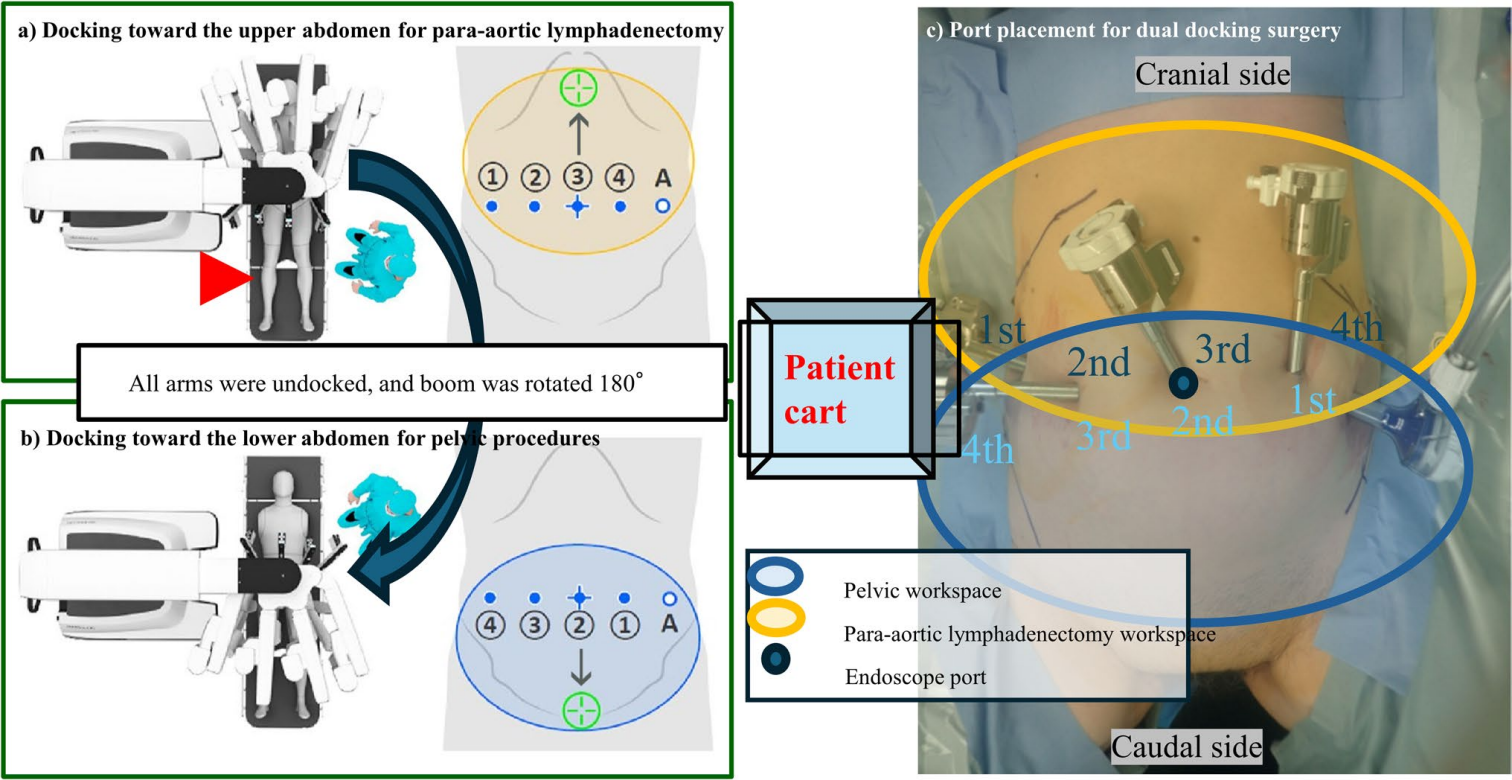
Dual robotic docking using the same ports in retroperitoneal lymphadenectomy. **a** Ports are placed laterally aligning at the level of the umbilicus. **b** Port placement for dual-docking surgery in endometrial cancer

- ***Procedure steps***
  i. Docking toward the upper abdomen
  ii. Para-aortic lymphadenectomy
  iii. Omentectomy (if indicated)
  iv. Undocking and re-docking toward the lower abdomen
  v. Pelvic lymphadenectomy
  vi. Hysterectomy (When cervical invasion was suspected, semi-radical hysterectomy was conducted, bilateral salpingo-oophorectomy
- ***Procedure details***
  i. Patients were placed in an open leg position or modified dorsal lithotomy position and examined in the Trendelenburg position (> 25°) adjusted for adequate exposure before docking. Antiskid methods (vacuum bean bags and gel pads) were used to avoid deviation from the Trendelenburg position.
  ii. Boom placement in upper abdomen and pelvic space procedures, and port placements are shown in Fig. 1a–c. The endoscope port was introduced through the umbilicus at the aorta bifurcation, and other ports were placed laterally aligned at the level of the endoscope port (Fig. 1a). Port placement was decided after proper insufflation. The endoscope was inserted, and the abdomen and pelvis were inspected.
  iii. Three Da Vinci ports and an assistant port (12 mm) were placed laterally, 7 cm from the initial endoscope port under direct vision. Da Vinci port distance should range between 6 and 10 cm according to habitus or internal anatomy.
  iv. Dock the 3rd arm to the endoscope port. Target the stomach’s greater curvature along the patient’s midline. Dock the 4th arm to the left of the endoscope port and 1st and 2nd arms to the right of the endoscope port.
  v. The initial procedural steps for para-aortic lymphadenectomy and omental resection (if indicated) were performed with the Xi system docked toward the upper abdomen.
  vi. After finishing the upper abdominal procedure, all arms were undocked, and the boom was rotated by 180°. The boom was approximately in line with the second target anatomy (Fig. 1b). There was no need to reposition or change the patient’s cart position.
  vii. Dock the 2nd arm to the endoscope port. Dock the 3rd and 4th arms to the right of the endoscope in the umbilicus, and the 1st arm the left of the endoscope port.
  viii. In the case of difficulty in retrieving the uterus transvaginally, it was placed in a retrieval bag and retrieved through a 12-mm port on the left side of the abdomen after undocking.

### Steps for para-aortic lymphadenectomy

i. Peritoneal incision just above the aortic bifurcation to confirm bifurcation.
ii. The peritoneum over the aorta is incised cephalad, the peritoneum is expanded laterally, and a straight needle is used to lift the peritoneum to form the surgical field.
iii. The lower para-aortic lymph node dissection is dissected in the order of right vena cava, left aorta, and arteriovenous (AV) intracavity. The right ureter and right ovarian AV vein are identified and dissected while avoiding injury to the lumbar vein.
iv. The higher para-aortic lymph nodes are dissected in the following order: left aortic, inferior vena cava, and AV intracavity. The right ovarian vein is cut with a vascular sealer at its confluence where it flows into the inferior vena cava. The left ovarian vein is cut at its confluence with the renal vein, and the caudal end of the left renal vein is cut with a vascular sealer because of the many lymphatic vessels and small blood vessels.

## Results

The characteristics of the 15 patients who underwent dual-docking robotic surgery for endometrial cancer are listed in Table 1. The median age was 55 years, median and mean ± standard deviation of body mass index (BMI) was 22.1 and 24.4 ± 4.57, and FIGO stage 1 was the most common estimated stage. None of the patients were preoperatively suspected to have extrauterine disease.

**Table 1:** Characteristics of all participants (*n* = 15)

Median (range) or number (%) as appropriate	No. of Patients (range or %)	
Age, years (range)	55	(28–72)
Height, cm (range)	157	(147–163)
Weight, kg (range)	59	(46–94)
* BMI (range)	22.1	(18–35)
Less than 20	3	(20%)
20–30	9	(60%)
over 30	3	(20%)
Vaginal delivery	0	(0–3)
No. of previous surgeries	1	(0–3)
Preoperative histological type		
Endometrioid	12	(80%)
Grade 1	8	(52%)
Grade 2	1	(7%)
Grade 3	3	(20%)
^†^SEIC	1	(7%)
Serous	1	(7%)
Carcinosarcoma	1	(7%)
Estimated^‡^ FIGO stage		
I A	3	(20%)
I B	9	(60%)
II	3	(20%)

*BMI, body mass index; ^†^SEIC, serous endometrial intraepithelial carcinoma; ^‡^FIGO, International Federation of Gynecology and Obstetrics

**Fig. 2:**
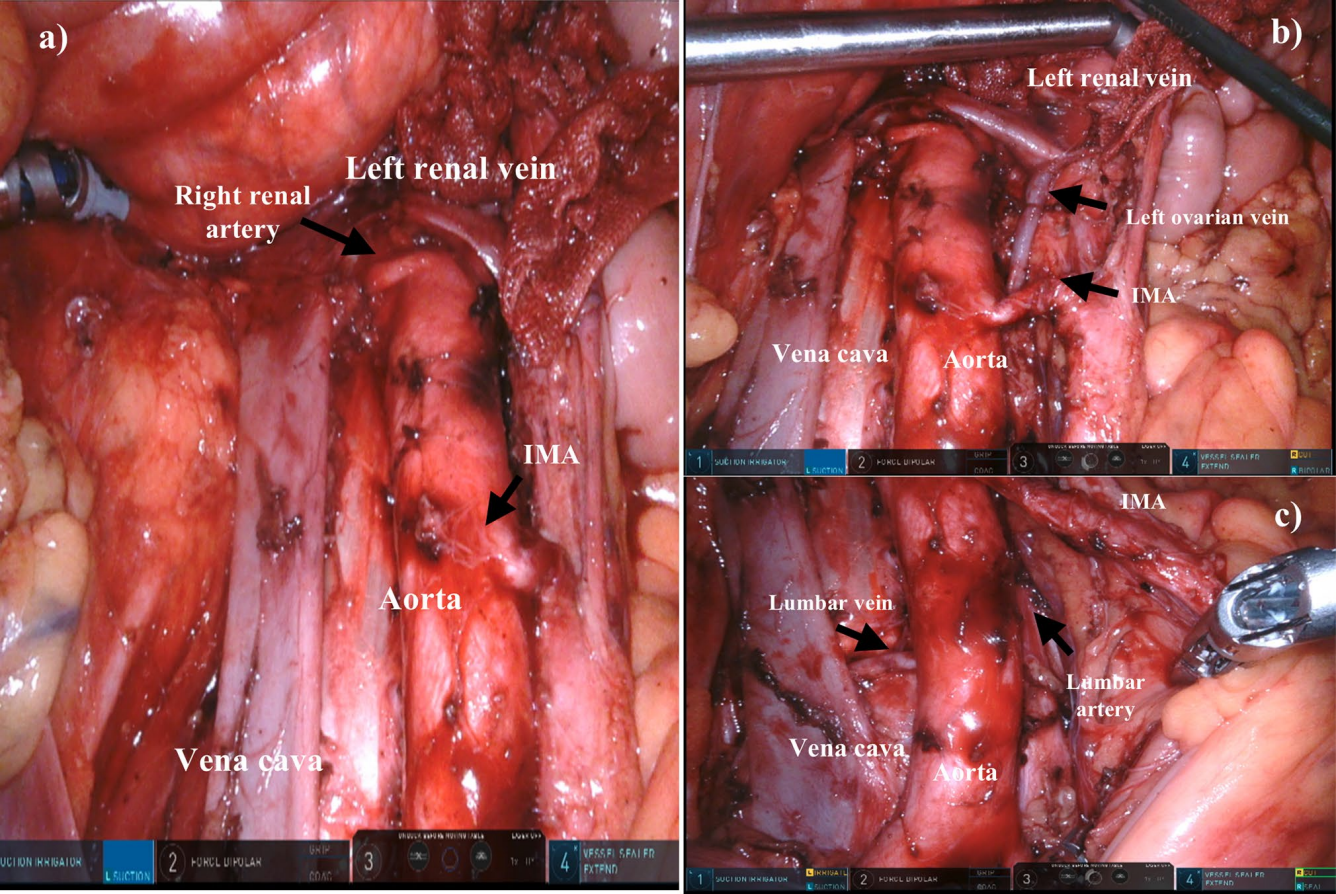
The photograph in Case 15 involving a para-aortic lymphadenectomy. **a** A photograph after para-aortic lymphadenectomy, **b** upper para-aortic lesion, **c** lower para-aortic lesion

Surgery was successfully completed robotically, including systemic lymphadenectomy, except in Case 11 which was converted to laparotomy. A photograph of the para-aortic lymphadenectomy in Case 15 is shown in Fig. 2.

Table 2 shows the surgical outcomes of dual-docking surgery in the 14 patients, and the results for each participant are shown in S1. Total extrafascial hysterectomy was performed in all cases except for four with suspected tumor cervical extension. None of the patients had para-aortic lymph node metastasis, and only Case 2 was positive for pelvic lymph nodes. The median operative times were almost identical between the upper and lower abdomen (183 and 206 min, respectively). The median rotation time was 17 min (range: 5–37 min), and gradually decreased, and Case 8 had the shortest at 5 min. Additional or unexpected port placement did not occur in any case. There were no significant differences in surgical outcome regarding patients with BMI < 20 compared to other patients. Perioperative complications are shown in Table 3. Intraoperative major complications included the following: In Case 6, laryngeal edema was caused by extended operation time to find a lost needle; in Case 11, right common iliac vein injury, which could not be controlled using robotic surgery, led to conversion to laparotomy; and in Case 14, right renal artery injury resulted in laparoscopic right nephrectomy following robotic gynecological procedures. Major postoperative complications included the following: In Case 1, a minor ureterovaginal fistula spontaneously cured without invasive treatment; a grade 2 pelvic infection in Case 4; and grade 2 chylous ascites occurred in three cases. In Case 13, resection of the rectal surface was performed intraoperatively, and lavage of the pelvic cavity for a pelvic abscess was performed laparoscopically on postoperative day seven. All complications resolved spontaneously and did not lead to serious complications. Three of the fifteen patients (Cases 2, 5, and 13) were unexpectedly diagnosed with advanced disease, and eleven of the fifteen patients underwent postoperative chemotherapy (Table S2). Only one patient with stage IVB (Case 13) recurrence was identified with 6 months of progression-free interval, within a median follow-up period of 54 months (range: 13–64 months).

**Table 2:** Surgical outcomes of dual docking robotic surgery (*n* = 14)*

Variable	Median or *n* (%) as appropriate	
Procedures for uterine resection		
Total extrafascial hysterectomy	10	(71%)
Semi-radical hysterectomy	4	(29%)
Other resected organs		
Omentum	5	(36%)
Appendix	2	(14%)
Number of lymph nodes		
Para-aortic (range)	19	(6–29)
Pelvic (range)	28	(15–42)
Blood loss, mL (range)	162	(20–685)
Operative times (range)	480	(371–834)
^†^Upper abdomen console times, min (range)	183	(105–370)
^‡^Rotation times, min (range)	21	(5–41)
^†^Lower abdomen console times, min (range)	206	(90–295)
Postoperative hospital stay (range)	9	(5–17)

*A case 11 was excluded because of convert to laparotomy

^†^The times between the surgeon sit on the console chair and finish the upper/lower console

^‡^The times between finish the upper console and start the lower console

**Table 3:** Perioperative complications by the Clavien-Dingo Classification version 2.0

Intraoperative			Postoperative			
Case		Grade*	Early phase	Grade*	Late phase	Grade*
1	Nil	Nil	Vaginal stump infection	II	Legedema Ureterovaginal fistula	I IIIa
2	Nil	Nil	Right leg pain Higher serum creatine kinase	I I	Nil	Nil
3	Nil	Nil	Shoulder, extremities pain Neurogenic bladder	I II	Legedema	I
4	Nil	Nil	Pelvic infection	I	Nil	Nil
5	Nil	Nil	Shoulder pain Chylous ascites	I II	Nil	Nil
6	Laryngeal edema	I	Higher serum creatine kinase Extremities numbness	I I	Nil	Nil
7	Nil	Nil	Urinary infection Chylous ascites	I II	Nil	Nil
8	Nil	Nil	Higher serum creatine kinase	I	Incisional hernia Asymptomatic lymphocyst	I I
9	Nil	Nil	Lymphatic retention	I	Legedema	I
10	Subcutaneous emphysema	I	Nil	Nil	Nil	Nil
11	*Right common iliac vein injury	IVa	Nil	Nil	Nil	Nil
12	Nil	Nil	Nil	Nil	Nil	Nil
13	Nil	Nil	^†^ Pelvic abscess	IIIb	Nil	Nil
14	^‡^Rt. renal artery injury	IVa	Higher serum creatinine Higher creatine kinase Chylous ascites	I I II	Nil	Nil
15	Nil	Nil	Nil	Nil	Nil	Nil

*convert to laparotomy; ^†^Laparoscopic lavage was performed, postoperative day7; ^‡^Laparoscopic right nephrectomy was underwent following robotic gynecological procedures

### The supplementary procedures in our standard treatment

The degree of inclination was 26° in the Trendelenburg position, which was the upper limit of our facility. Patients beginning with Case 4 were postured in an open leg position, changed from a modified lithotomy position, and shoulder-holding devices were removed to avoid pain in the extremities. The only antiskid method used was a pink pad between the bed and back. Consequently, the endoscope port was placed 2 cm above the umbilicus in Case 1, and the port placement for all other patients was at the level of the umbilicus. Complete lateral alignment of the ports was achieved in all patients. The spacing of the lateral ports was 6–8 cm apart, even in three lean patients, and this was extended to 10 cm in an obese patient. These lateral ports were placed in the assistant port (12 mm) (Cases 4–15) on the outermost part of the Da Vinci port at a lateral distance of 7 cm. The ventral port was located in the middle of the inferior rib margin and iliac crest. The Endowrist^®^ instruments (Intuitive Surgical, Inc.) procedure stated the following for upper abdominal procedures: Endowrist^®^Vessel Sealer in the 4th arm, Endoscope in the 3rd arm, Monopolar Curved Scissors^®^ in the 2nd arm, Maryland Bipolar Forceps^®^ in the 1st arm; and for lower abdominal procedures: Maryland Bipolar Forceps^®^ in the 1st arm, Endoscope^®^ in the 2nd arm, Monopolar Curved Scissors^®^ in the 3rd arm, and Endowrist^®^Vessel Sealer in the 4th arm for lower abdominal procedures. Other instruments were used, including the Cadiere Forceps^®^ for grasping, a Large Needle Driver^®^, and a Mega SutureCut^®^ Needle Driver for needle driving.

## Discussion

We found robotic dual-docking surgery performed with the ports placed laterally aligned at the level of the umbilicus to be applicable to endometrial cancer. Using our standard protocol, we achieved almost complete surgical staging without the need for additional port placement. The number of lymph nodes, including para-aortic lymphadenectomy up to the left renal vein level removed by our system, was the 2nd highest among other studies [6, 7, 11–16, 19–21], ranging between 19 and 52, and only one patient experienced a recurrence of the disease, with no deaths being reported during the long-term follow-up periods. The relatively long console time in our cohort may be attributed to extensive lymph node dissection.

Initial reports regarding robotic surgery for para-aortic lymphadenectomy in endometrial cancer were published in 2008 [5–7]. A literature review, including a feasibility assessment via the intraperitoneal approach is shown in Table 4. Evidence from Asia, especially Japan, has not yet been reported; however, our analysis, following an adequate period of observation, suggests that that this procedure be adopted as a surgical standard.

**Table 4:** The literatures of the transperitoneal para-aortic lymphadenectomy mainly involving intermediate to high-risk endometrial cancer

Authors	Year (y)	Trial	Da Vinci System	Trocar placements		Number of patients	BMI (Median)	Operation times (min, Median)	Blood loss (mL, Median)	Number of transfusion patients	Number of lymph nodes resection (median)		Number of conversion	Perioperative complications	Post-operative complications
				Camera port	Other ports						Pelvic	Para-aortic			
Seamon et al. [5]	2008	Prospective	Da Vinci or H	not listed	not listed	105*	34	N/A	99	N/A	21	9	13	One vena cava injury, two gastrointestinal events	One pneumina, one hemorrhage, seven colitis, one chest pain, one vaginal leakege, one pelvic abcess, one seroma
DeNardis et al. [6]	2008	Retrospective	Da Vinci	approximately 22 to 26 cm above the pubic symphysis (2 to 6 cm above the umbilicus)	Two were placed at the lateral borders of the rectus sheath. An additional 8 mm was inserted 10 cm lateral and just superior to the left robotic port	56	28.5	177 (Mean)	105 (mean)	0	13 (mean)	6.5 (Mean)	3	Two persistent bleeding	One ileus, one respiratory failure, one wound infection, four vaginal cuff hematoma, one urinary tract infection
Gehrig et al. [7]	2008	Retrospective	Da Vinci	not listed	not listed	45	37.5	189 (Mean)	50 (Mean)	N/A	21	10	0	None	Two port-site herna, one lymphedema, one enterotomy, one vaginal cuff complication
Magrina et al. [12]	2010	Retrospective	Da Vinci or Si	One in umbilicus, one in 2 or 3 fingerbreadths suprapubically and 1 or 2 fingerbreadths to the left of the midline	10 cm to 12 cm to the lateral of camera port	6	26.3 (mean)^†^	N/A	45 (mean)^†^	None	N/A	12.9 (mean)^†^	1	One aorta bleeding, one active bleeding†	One lymphcyst formation†
Cardenas-Goicoechea et al. [19]	2010	Retrospective	Da Vinci	Approximately 25 cm above the pubic symphysis	The three accessory robotic ports placed laterally at 8–10 cm intervals, and the fifth port on the right flank, in line with the other ports	102	32.3	222	100	3	20	9	1	Two Intestinal tract	One pulmonary embolism, one enterocuatneous fistula
Zanagnolo *et al. *[13]	2013	Retrospective	S or Si	One in umbilicus, one in 3 or 4 cm suprapubically and 1 or 2 cm to the left of the midline	10 cm to 12 cm to the lateral of camera port	6	23 (mean)^‡^	285†	50 (mean)^‡^	3†	15 (mean)^‡^	14 (mean)^‡^	None	Two significant bleeding (> 500 mL)‡	Seven chlous ascites, two vaginal leakages, one ureteral fistula, one femoral nerve injury, four leg edema, two port-site hernias, four lymphoceles, one lymphaic ascites‡
Franke et al. [14]	2015	Retrospective	Si	One in umbilicus, one in hypogastric area	A curve line	19	25.1	326	178	None	N/A	10.9	None	None	None
Ekdahl et al. [16]	2016	Retrospective	Si	In the midline at the level of iliac spine	Two in lateral of camera port, one in 8 cm right side of umbilicus	76^§^	26^§^	220^§^	100^§^	N/A	20	16	1	One vaginal deceleration	One abdominal abcess, one chlous ascites, two port site hernias, one ureteral injury, and one small intestine injury
Lee et al. [20]	2018	Retrospective	S or Xi	Umbilicus	Lateral of camera port	26	25.4	N/A	105	N/A	29	23	N/A	One aorta injury, one pulmonary embolism and one rebleeding	None
Loaec *et al. *[15]	2018	Retrospective	Si	One in umbilicus, one in median supra-pubic	Laterally	20	24	240	N/A	N/A	11	19	2	One urinary injury	One small bowel incarceration (reoperation) One vaginal vault abcess
Bebia et al. [21]	2021	Prospective	S or Xi	One in umbilicus, placement was changed depending on the technique		38	30.8	261	100	2	N/A	10	1	vascular injury, inferior meseteric artery	None
Present study	2024	Prospective	Xi	2 cm above in the midline at the upper anterior iliac spine level	Lateral of camera port	15	22.1	500.5^||^	195^||^	One	28^||^	19^||^	1	One rt. common iliac vein injury and rt. renal artery injury	Two extremities pain, two chylous ascites, one pelvic infection, one deep venous thrombosis

N/A not applicable

*Pelvic and paraoartic lymphaenectomy was performed in 81/105 patients

^†^Include malignancies with: ovarian; 20 patients, endometrial; six patients, cervical; four patient, vaginal; one patient and peritoneal; two patient

^‡^Include malignancies with: ovarian; 40 patients, endometrial; six patients, tubal; four patients, cervical; one patient

^§^Including seven patients with aborted para-aortic lymphadenectomy

^||^Excluding one patients with converted laparotomy

Para-aortic lymphadenectomy as a dual-docking surgery was conducted by Magrina et al*.* [12] in 2010, but these procedures were not completely dual-docked robotic systems because of the need to turn the patient’s bed and the requirement for additional port placement. Meanwhile, Zanagnolo et al*.* [13] report a small alteration to their robotic surgery using the Da Vinci S or Si system, while Pakish et al*.* [22] describe the utility of a retroperitoneal approach, in which more para-aortic lymph nodes could be resected than during a transperitoneal approach (the median number of lymph nodes: 10 in retroperitoneal approach vs. 4 in transperitoneal approach). However, the range of arm motion of transperitoneal approach is limited, and unlike laparoscopic surgery, robotic surgery requires repositioning or changing the patient’s cart position. Therefore, we consider that dual-docking surgery is more realistic. The usefulness of double-docking compared with single-docking is described by Franke et al*.* [14]. In double docking for para-aortic resection, one endoscope port and three other ports are located in the lower abdomen under the Da Vinci Si system docked on the patient’s head. In pelvic lymphadenectomy following para-aortic lymphadenectomy, the system was undocked, the patient’s cart was moved between the two legs, and the endoscope port was placed on the umbilicus. Although the double-docking technique leads to approximately twice the number of resected lymph nodes compared to single-docking, the surgical time is quite long, and docking is complex.

We introduced the lateral alignment of the ports, and a similar retrospective study of port placement was conducted by Ekdahl et al*.* [16]. The endoscope port in double-docking surgery using the Da Vinci Si system was introduced laterally at the level of the iliac spine, which was almost 3 cm caudal to the umbilicus. After the upper abdominal procedures on the patient’s right side above the robotic column, the column was rotated, and the patient’s bed was moved appropriately for the lower abdominal procedures. Similarly, Loaec et al*.* [15] report the use of an endoscope port placed at the median suprapubic and umbilicus for para-aortic lymphadenectomy in pelvic lymphadenectomy.

Using the latest Da Vinci Xi system, we have developed some newer improvements including: (1) docking from any direction by turning the boom, (2) the endoscope port can be attached to any arm since it has been miniaturized to 8 mm in diameter, and (3) the patient clearance function and enlargement of the range of motion and distance by the end-list forceps which are 4.5 cm longer than Si, made it possible to laterally align four Da Vinci 8 mm ports during dual-docking surgery. These improvements have led to the ease of dual-docking, and in most situations when using the Da Vinci Xi system, the patient cart or bed does not need to be moved.

A recent retrospective report by Lee et al*.* [20] compares the Da Vinci S system and laparoscopic surgery with a robotic endoscope port fixed at the umbilicus regardless of the patient’s habitus, with the other ports aligned at the level of the umbilicus. Previous studies have been conducted based on earlier robotic systems, and there is a lack of evidence including prognostic outcomes for prospectively evaluating dual-docking surgeries using the Da Vinci Xi system.

The advantages of robotic surgery for laparotomy or laparoscopy in obese patients with endometrial cancer have been described [7, 11, 23, 24]. Laparoscopic surgery in obese patients results in fewer postoperative complications, shorter hospitalization, and lower levels of postoperative pain than laparotomy [7, 23]. Compared to less invasive surgical approaches, a robotic surgery patient group that had a significantly higher BMI showed superior surgical outcomes over a laparoscopy group who underwent comprehensive surgical staging [11]. Regarding the degree of obesity in patients undergoing robotic surgery, BMI classification did not correlate with conversion to laparotomy or complication rate, and node dissections were equivalent in the three categories [24].

The novelty of our dual-docking surgery is that it provides a suitable port placement arrangement. Changing the surgical range from the upper abdomen to the lower abdomen can be simply performed by rotating the boom without moving the patient cart and by directly docking the ports in the Da Vinci Xi system. No complications due to the most frequently used Endowrist^®^ Vessel Sealer were found in retroperitoneal lymphadenectomy or in the difficulty of robotic operation. However, 4 out of 15 patients (26.7%) experienced major complications. The injury to the right common iliac vein in Case 11 was not attributable to a technical error but rather occurred during rapid forceps manipulation. No further injuries were observed after this case because the standard technique for para-aortic lymph node dissection described in “Materials and methods” was fully established and carefully performed. The reason for the right renal artery injury in Case 14 may have been that the camera could easily reach the deep field under the renal vein and provide a view different from that of laparotomy. Although these complications are specific to endoscopic surgery, they are not technical problems that occurred in only a single case; therefore, they do not account for the complexity of this technique.

Robotic endometrial cancer surgery in lean Asian patients has not yet been described, and our cohort is the leanest recorded in the literature (Table 4) [5–7, 12–16, 19–21]. We verified that for certain body habitus types, the positioning of ports allowed for successful surgery without interfering with the movement of intraoperative forceps. Three patients with a BMI greater than 30 underwent surgery successfully without experiencing serious perioperative complications. In addition, a BMI of less than 20 allowed for the placement of a laterally aligned port that accommodated robotic forceps. The analyzed surgical outcomes of the literatures and our study are shown in S3. The number of lymph nodes for para-aortic lymph node dissection was positively correlated with operative time and conversion rates, with a higher number of lymph nodes removed in our facility and longer operative time. Contrary to expectations, the number of para-aortic lymph node dissection decreased as BMI increased, with our cohort being the leanest and most adequately dissected. In the learning curve of our facility, as the number of experienced cases increased, operating time tended to decrease. Our study was limited by the small number of patients and the inclusion of a large range of body types. Although the surgeon performing all procedures was experienced, the median surgical time, especially for para-aortic lymphadenectomy, was longer than that reported elsewhere. Approximately 20 procedures are needed for a surgeon to become proficient in performing retroperitoneal lymphadenectomy for endometrial cancer [5]. Most of our cases were successful, but we also experienced three cases with serious complications in dual-docking surgery, indicating that the results of our study require further research.

In conclusion, this technique allowed enough lymph nodes to be dissected in patients of various types of habitus including lean common among the Japanese, and the prognosis was excellent. This study demonstrated that dual-docking surgery, facilitated by the introduction of the Da Vinci Xi system, has the potential to become a standard procedure for robotic endometrial cancer surgery. Further extensive clinical trials on the feasibility of dual-docking surgery are needed.

## Supplementary Information

Below is the link to the electronic supplementary material.

Supplementary file 1 (DOCX 22 KB)

Supplementary file 2 (DOCX 16 KB)

Supplementary file 3 (TIF 2005 KB)

## Data Availability

The data used in this study are available upon reasonable request by writing to the corresponding author.
