# Supplementary material for: Robotic dual-docking surgery for para-aortic lymphadenectomy in endometrial cancer: a prospective feasibility study
Source: Int J Clin Oncol. 2024 Dec 21;30(2):358–70. doi: 10.1007/s10147-024-02635-8 (PMC11785595; doi:10.1007/s10147-024-02635-8)
Supplement: Supplementary file 1 — (DOCX 22 KB) [file 10147_2024_2635_MOESM1_ESM.docx]

| Supplemenatary 1 Details of the surgical outcome of the participants (n=15) | | | | | | | | | | | | | |  |  |  |
| --- | --- | --- | --- | --- | --- | --- | --- | --- | --- | --- | --- | --- | --- | --- | --- | --- |
| Case | BMI | Type of hysterectomy | Other resected organs | Position | Port | Operating -time (min) | console times, min | | Blood loss, ml （range） | Hospital stay（range） | Number of resected lymph nodes | | Conversion | Postoperative laboratory data (maximum) | | |
|  |  |  |  |  |  |  | Upper | Lower |  |  | Para-Aorta | Pelvis |  | Creatinine kinase (IU/L) | C-reactive protein (mg/dL) | White blood cells (/μL) |
| 1 | 21.0 | Semi-RAH |  | modified dorsal lithotomy | Two rows | 545 | 105 | 295 | 150 | 10 | 25 | 34 | Nil | 4471 | 18.33 | 17820 |
| 2 | 24.7 | Semi-RAH |  | modified dorsal lithotomy | Two rows | 658 | 141 | 370 | 320 | 9 | 19 | 42 | Nil | 978 | 10 | 8450 |
| 3 | 30.9 | Simple | - | modified dorsal lithotomy | Two rows | 452 | 122 | 198 | 40 | 9 | 25 | 32 | Nil | 1132 | 7.27 | 7600 |
| 4 | 19.1 | Simple | Omentum,appe | Open leg | Two rows | 427 | 144 | 195 | 30 | 16 | 14 | 23 | Nil | not measured | 17 | 14450 |
| 5 | 24.9 | Simple | Omentum | Open leg | Align | 587 | 323 | 148 | 175 | 8 | 12 | 18 | Nil | 140 | 6.13 | 5660 |
| 6 | 26.2 | Simple | - | Open leg | Align | 834 | 303 | 333 | 445 | 8 | 25 | 25 | Nil | 1421 | 6.68 | 12610 |
| 7 | 19.4 | Simple | - | Open leg | Align | 456 | 203 | 188 | 215 | 8 | 15 | 22 | Nil | 552 | 6.02 | 10060 |
| 8 | 36.8 | Simple | - | Open leg | Align | 638 | 203 | 312 | 685 | 11 | 14 | 15 | Nil | 3367 | 12.04 | 8740 |
| 9 | 21.2 | Semi-RAH | Omentum | Open leg | Align | 545 | 203 | 215 | 150 | 17 | 23 | 32 | Nil | not measured | 5.9 | 1396 |
| 10 | 22.1 | Simple | Omentum | Open leg | Align | 371 | 175 | 120 | 20 | 7 | 29 | 36 | Nil | 371 | 2.94 | 7070 |
| 11 | 20.1 | Semi-RAH |  | Open leg | Align | 440 |  |  | 4570 | 10 |  |  | To laparotomy |  |  |  |
| 12 | 34.5 | Simple |  | Open leg | Align | 437 | 159 | 228 | 130 | 5 | 6 | 39 | Nil | 532 | 9.68 | 8130 |
| 13 | 24.5 | Simple | dissemination resection | Open leg | Align | 455 | 149 | 237 | 80 | 11 | 19 | 18 | Nil | 152 | 41.7 | 14270 |
| 14 | 21.9 | Simple | Lt. kidney | Open leg | Align | 504 | 338 | 90 | 302 | 14 | 24 | 24 | Rt nephrectomy following surgery | 651 | 5.4 | 8850 |
| 15 | 18.9 | Simple | Omentum,appe | Open leg | Align | 448 | 192 | 191 | 302 | 7 | 22 | 26 | Nil | 154 | 2.81 | 6180 |
| RAH, radical hysterectomy | | | | | | | | | | | | | |  |  |  |
