# Supplementary material for: Robotic dual-docking surgery for para-aortic lymphadenectomy in endometrial cancer: a prospective feasibility study
Source: Int J Clin Oncol. 2024 Dec 21;30(2):358–70. doi: 10.1007/s10147-024-02635-8 (PMC11785595; doi:10.1007/s10147-024-02635-8)
Supplement: Supplementary file 2 — (DOCX 16 KB) [file 10147_2024_2635_MOESM2_ESM.docx]

| Supplementary 2 Prognostic outcomes of participants (n=15) | | | |
| --- | --- | --- | --- |
|  | Variables | | No. of Patients (%) |
|  | Post-operative histological type | |  |
|  |  | Endometrioid | 12 (80.0%) |
|  |  | Grade 1 | 8 (54%) |
|  |  | Grade 2 | 2 (13%) |
|  |  | Grade 3 | 2 (13%) |
|  |  | SEIC | 1 (7%) |
|  |  | Serous | 1 (7%) |
|  |  | Carcinosarcoma | 1 (7%) |
|  | *FIGO stage | |  |
|  |  | I A | 6 (40%) |
|  |  | I B | 3 (20%) |
|  |  | II | 3 (20%) |
|  |  | III | 2 (13%) |
|  |  | IV | 1 (6%) |
|  | Adjuvant therapy | |  |
|  |  | None | ^†^4 (27%) |
|  |  | Chemotherapy | 11 (73%) |
|  | Recurrence | |  |
|  |  | None | 14 (93%) |
|  |  | Yes | 1 (7%) |
|  | Death | |  |
|  |  | None | 15(100%) |
|  |  | Yes | 0 |
| * FIGO, International Federation of Gynecology and Obstetrics (FIGO, 2008) | | | |
| ^†^ Including one patient with rejected adjuvant chemotherapy | | | |
