# Supplementary figures and images for: Robotic dual-docking surgery for para-aortic lymphadenectomy in endometrial cancer: a prospective feasibility study
Source: Int J Clin Oncol. 2024 Dec 21;30(2):358–70. doi: 10.1007/s10147-024-02635-8 (PMC11785595; doi:10.1007/s10147-024-02635-8)

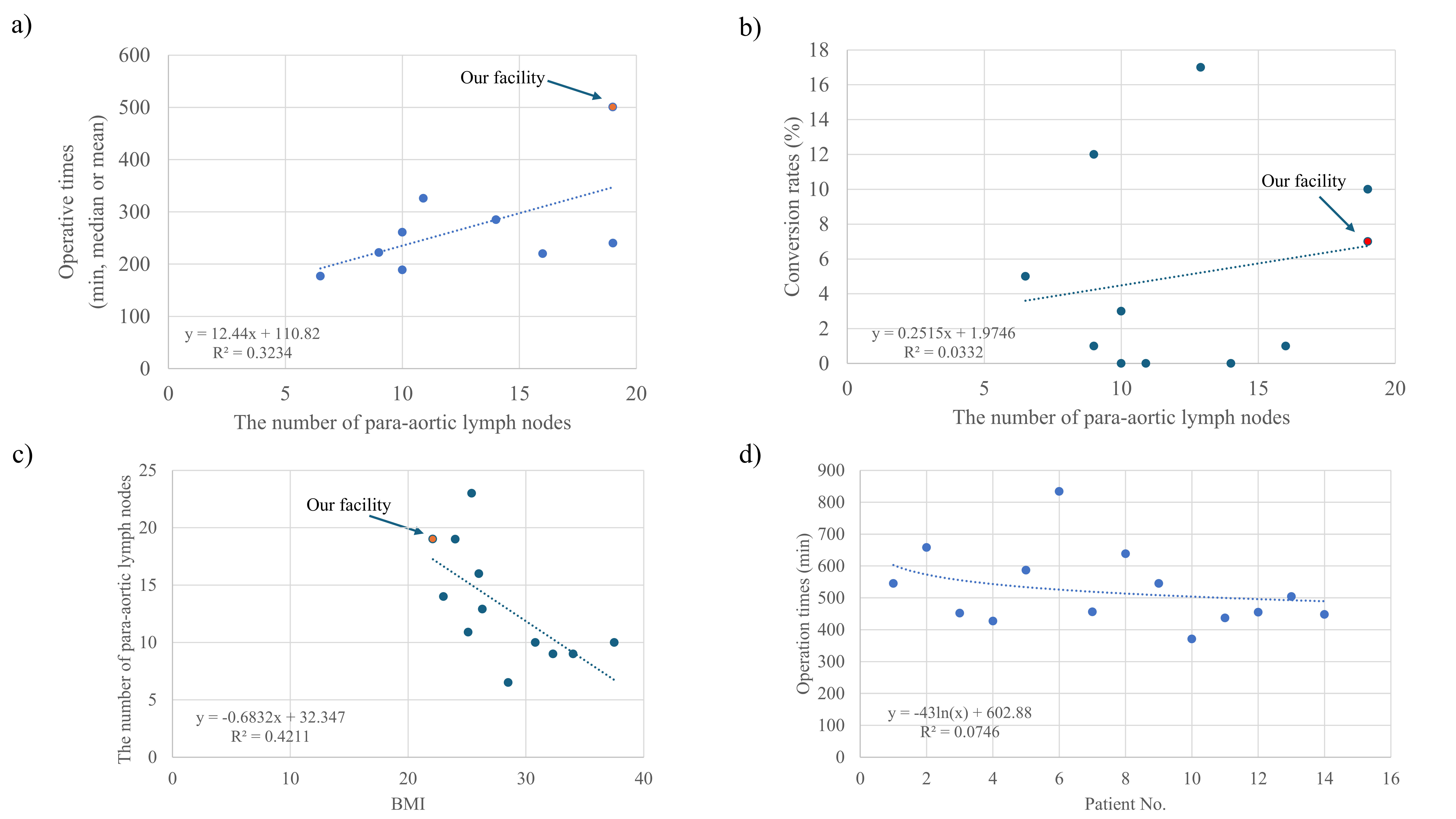

Supplement: Supplementary file 3 — (TIF 2005 KB) [file 10147_2024_2635_MOESM3_ESM.tif]
